# Editorial: Cardiovascular involvement during sepsis: therapeutic and prognostic consequences

**DOI:** 10.3389/fcvm.2023.1314834

**Published:** 2023-11-01

**Authors:** F. Innocenti, V. Palmieri

**Affiliations:** ^1^ High-Dependency Unit, Department of Clinical and Experimental Medicine, Azienda Ospedaliero-Universitaria Careggi, Firenze, Italy; ^2^ Cardiosurgery Unit, Cardiovascular Department, “Sant’Anna e San Sebastiano” National Hospital, Caserta, Italy

**Keywords:** sepsis, prognosis, resuscitation, septic cardiomyopathy, treatment

**Editorial on the Research Topic** Cardiovascular involvement during sepsis: therapeutic and prognostic consequences

Defining the moment when an infection evolves into sepsis can be a difficult task, as there are no clear symptoms or biomarkers for clinicians to rely on. When clinical conditions deteriorate and the patient requires medical intervention, symptoms may appear ambiguous and not correlate with the severity and risk of progression, as it often occurs with referred fatigue that rapidly evolves in to cardiocirculatory shock.

In the presence of sepsis, hypotension may be the manifestation of a number of pathophysiological processes running in parallel (1). At the vascular level, a cytokines storm as well as the secretion of multiple vasoplegic substances, like nitric oxide, determines a reduced arterial and venous tone. The endothelial dysfunction, with fluid leakage in the extravascular space, coupled with a possible reduced intake or increased loss of fluids in the first days of the infection, contribute to determine a condition of absolute and relative hypovolemia. An acute myocardial dysfunction (sepsis-induced myocardial dysfunction, SIMD) develops in up to 80% of septic patients and can involve systolic and/or diastolic function of both ventricles.

In actuality, diagnosing the relative weight of the aforementioned mechanisms remains difficult, considering that up to 70% of sepsis cases are diagnosed in the Emergency Department. The treatment usually begins based on the measure of blood pressure and our attention is captured by systolic blood pressure. It represents an index of cardiac afterload and has to be maintained at >90 mmHg by the administration of fluids and vasopressors. In the absence of feasible methods to measure arterial tone, we could reconsider diastolic blood pressure (2). It correlates with the severity of arteriolar vasodilation and represents tissue perfusion pressure, especially for the coronary district, which fills only during diastole. This measure is often neglected during the early resuscitation of septic patients, but it could integrate both the initial evaluation and the titration of vasopressor medications. The only drawback of this parameter is the need for an invasive monitoring of blood pressure in order to have an accurate evaluation, but this is possible even in an Emergency Medicine setting. The clinical value of central venous pressure as an index of preload adequacy is dampened by its multifactorial determinants, which make it an inconsistent measure of right ventricle telediastolic volume, coupled with the difficulty to obtain a central venous line in the very early stages of resuscitation.

However, the presence of SIMD was an unexpected find in critical care medicine, where the classical view of the cardiac involvement in septic shock consisted of a high-cardiac output state, with low systemic resistances. The advent of transesophageal and transthoracic echocardiography, and their growing employment in ICUs, allowed clinicians to ascertain the presence of myocardial dysfunction in the majority of septic patients (3). The possibility to diagnose early the presence of a SIMD would be considerable progress towards a comprehensive assessment at the beginning of resuscitation, but, in actuality, it does not seem a currently feasible objective.

A continuously growing interest for this aspect of the septic syndrome is acknowledged by the bibliometric analysis by Yao and coll. included in the RT (Yao et al.). While initial studies focused on epidemiological aspects, more recent papers have dealt with the molecular mechanisms of SIMD, especially oxidative stress and regulated cell death, which could become therapeutic targets in the future.

A relevant issue is represented by the fact that the diagnosis of SIMD is actually based on the echocardiographic examination, and the expertise required goes far beyond that necessary to perform the point-of-care examination, which is so widely employed in the Emergency Medicine setting. The disease may present with different characteristics and, as outlined by Shvilkina and Shapiro, measurement of the left ventricular ejection fraction does not give an accurate evaluation as it is a parameter heavily influenced by pre- and afterload, and so is deeply altered by the septic process itself. The evaluation of Global Longitudinal Strain, an index of contractility relatively independent to loading conditions, even though it is derived from conventional echocardiographic views, may be difficult to employ in the early stages of resuscitation.

The need to better characterize the relative weight of the vasoplegic and myocardial dysfunction component has probable consequences on the early management of sepsis, as outlined in the aforementioned paper. Most of the treatments employed in the early resuscitation of septic patients are supported by low-quality data and represent hot topics in the scientific community. The introduction of new practices, like a restrictive regimen for fluid administration or the early administration of vasopressors, although with a meaningful rationale, did not bring about the expected results. In fact, most of the trials did not show a significant prognostic advantage over current therapeutic practices. We wonder if, beyond these disappointing results, there is an inadequate selection of patients, based on the actual absence of reliable criteria to distinguish those with prevalent reduction of the arterial tone from those who present a relative hypovolemia or impaired myocardial function.

The investigation into the possible role of genetic factors, as in the paper by Li et al., will give insights for a potential new therapeutic approach. The authors identified THBS1 as a key immune-related gene, with a potential role as a biomarker for the diagnosis of SIMD, as well as in the regulation of the pathogenesis of SCM.

Ideally, we would take advantage of biomarkers in sepsis, as “troponin” has helped create an early and reliable diagnosis of acute coronary syndrome in specific cases. Lipocalin, a key regulator of lipid metabolism, has been proposed by Liu et al. The multiplicity of systems activated during sepsis, which encompasses the immune response, the coagulation cascade, vasoactive substances, and metabolic mediators, is probably one of the reasons why it is so difficult to find a single and reliable biomarker to support the diagnosis of sepsis and, even more, of SIMD.

A further field of research is the reversibility of SIMD, as outlined in the papers by Owen et al. and Yan et al. The prolonged and intense inflammatory activation can determine an impairment of myocardial function, independent of usual cardiovascular risk factors. It can persist beyond the acute phase of the disease, due to a sort of tissue remodeling caused by persistent low-grade inflammation.

In conclusion, septic patients are very complex, and a global vision has to be maintained from the earliest phase of the illness in order to consider all the elements that could guide our therapeutic approach, without appealing to unrealistic attempts of oversimplification.
